# Psychosocial predictors of sexual initiation and high-risk sexual behaviors in early adolescence

**DOI:** 10.1186/1753-2000-1-14

**Published:** 2007-11-22

**Authors:** Argyro Caminis, Christopher Henrich, Vladislav Ruchkin, Mary Schwab-Stone, Andrés Martin

**Affiliations:** 1 Yale School of Medicine, Yale University, New Haven, CT, USA; 2 Department of Psychology, Georgia State University, Atlanta, GA, USA; 3 Center for Violence Prevention and Skonviks Psychiatric Clinic, Karolinska Institute, Sweden; 4 Yale School of Medicine Child Study Center, Yale University, New Haven, CT, USA; 5 Yale School of Medicine Child Study Center, Yale University and Children's Psychiatric Inpatient Service, Yale-New Haven Hospital, New Haven, CT, USA

## Abstract

**Background:**

This longitudinal study examined psychosocial factors associated with risky sexual behavior in early adolescence.

**Methods:**

Data were collected through a self-report survey, the Social and Health Assessment (SAHA), which was administered in three waves between 2001 and 2003 to a cohort of incoming sixth grade students in the public school system (149 classes at 17 middle and high schools, N = 1,175) of a small northeastern city in the United States.

We first examined whether internalizing and externalizing problems in sixth grade, and the rate of change in these factors during middle school, were predictive of sexual initiation two years later, when most of the sample was in eighth grade. We then assessed whether internalizing and externalizing problems in sixth grade, and the rate of change in these factors during middle school, were predictive of engaging in high risk sexual behavior over the subsequent two years.

**Results:**

Externalizing factors are more predictive of sexual risk in early adolescence than are internalizing factors. Specifically, substance use and violent delinquency over the course of middle school were associated with higher, while anxiety with lower, sexual initiation rates during middle school. Additionally, increased substance use over the course of middle school was associated with greater likelihood of engaging in high risk sexual behavior.

**Conclusion:**

By identifying particular psychosocial risk factors among young adolescents, the findings of this study have implications for designing multi-dimensional programs aimed at preventing health-compromising sexual behavior among young teens.

## Background

Teenage pregnancy rates in the United States have declined since the early 1990s [1]. This trend is thought to be partly a function of more consistent contraceptive use and later onset of sexual activity than in previous years [2]. In 2002, 13% of females and 15% of males ages fifteen to nineteen reported having had sex before age fifteen, as compared to 19% and 21% respectively in 1995 [1,3]. While an encouraging trend, these statistics mask higher prevalence of early and high risk sexual activity among certain populations. For example, the average age of sexual debut among inner-city youth is thirteen years of age, three years earlier than the national average [4]. Additionally, African-American teens tend to initiate sex earlier than Caucasian or Latina teens, and are more likely to initiate prior to age thirteen than are Caucasian teens [4]. Earlier sexual debut among minority populations contributes to ongoing health disparities, with rates of HIV, other sexually transmitted diseases (STDs), and unintended pregnancies disproportionately high among minority adolescents [5].

Previous studies have found that initiating sexual activity before age sixteen increases the likelihood of having an unintended pregnancy, inducing pre-cancerous changes in the cervix, and contracting STDs, including HIV/AIDS [5-12]. In spite of decreased teen pregnancy rates, 11% of all US births are among teenage girls and the teen pregnancy rate in the United States is two to eight times that of many other developed countries [13,14]. Of new STD infections each year, 48% are among people ages fifteen to twenty-four years old [15]. These consequences affect not only the adolescents themselves, but can incur a high cost to society through the need to support adolescent childbearing and its contribution to infant mortality [16].

In contemporary American society, what was once considered an early adulthood transition has arguably been evolving over the past several decades into anticipated behavior in middle and late adolescence [17]. The links between sexual intercourse and numerous psychosocial factors, including substance abuse, low self-esteem, depression and suicide attempts have been found to be strongest among younger adolescents in the United States [18]. Sexual intercourse may represent a marker of psychological distress when it occurs early, as opposed to at a more normative time [18]. These findings speak to the vulnerability of young adolescents and the importance of examining the relationship between psychosocial factors and early sexual behavior.

Whereas correlations between demographic factors and adolescent sexual activity have been fairly robust in the literature, psychological and behavioral correlates of early sexual behavior are less well understood [12,19]. This study is part of a larger research project related to adolescent development and is the second in a study of psychosocial risk factors associated with sexual onset among young urban, minority teens. In a cross-sectional study examining the sexual behavior of young adolescent girls, we found that sexually active girls under age sixteen endorsed significantly more symptoms of depression, had a more pessimistic outlook of their futures, felt less academically motivated, and did less well in school than those who were not sexually active [20]. The current longitudinal study expands on that first study by including both genders and by examining potential causal links between hypothesized psychosocial risk factors for early and high risk sexual activity. Prospective studies such as this can help to identify incipient factors exerting influence over adolescent development [21] and also potentially improve interventions to reduce such health-compromising behaviors.

Given consistent associations between demographic factors and early sexual behavior, we have controlled for certain factors including socio-economic status (which includes family structure, parental education, and a proxy measure for economic status), peer pressure, and sensation-seeking behavior. Previous studies have shown that children who live with both biological parents are less likely to be sexually active than those from one-parent homes, that increased maternal education is associated with later age of adolescent first intercourse, and that as socio-economic status decreases, rates of sexual activity tend to increase [22]. Other studies have examined peer influences on sexual initiation, concluding that perceived degree of peer sexual activity is directly related to adolescent sexual behaviors [23-26]. Likewise, sensation seeking behavior, defined as the tendency to pursue novel and stimulating experiences [27], has been reported as a factor also presumed to antecede sexual activity [23,24,28] We maintained separate variables for both genders in order to determine moderating effects of gender on the variables of interest.

Our study is informed by a conceptual framework that emphasizes the reciprocal relationship between three systems of influence on adolescent sexual behavior, including the self system, the familial system and the extra-familial system [19]. We focus on the correlations between teenage sexual behavior and two variables within the self system, namely psychological and behavioral factors. To study the psychological and behavioral correlates of risky adolescent sexual activity, we have used an additional conceptual framework adopted from the field of child psychology which distinguishes between 'externalizing,' or disorders characterized by behavioral disinhibition (disruptive behavior disorders of childhood) and 'internalizing,' or disorders characterized by negative mood states and inhibition (depression, anxiety) [21,29]. Our study seeks to apply this conceptual framework to examine how engaging in sexual risk behavior is influenced by internalizing factors, including depression, anxiety, and post-traumatic stress and by externalizing factors, including substance abuse, violent and non-violent delinquency.

Previous studies have identified several externalizing (or behavioral) and internalizing (or emotional) psychosocial factors influencing risky sexual behaviors among adolescents [4]. In terms of externalizing behaviors, studies have examined teen sex in the context of sociological literature on "deviant behavior" (which is understood as behaviors which depart from the regulatory norms of conventional society defining appropriate behavior for that age or stage in life) [22,30]. Some researchers have suggested that correlations between deviance and sexual behavior may be even stronger for younger initiators given that sexual intercourse at an earlier age is considered more deviant behavior than when it occurs at a more normative time [17]. A theory elaborated by [31] regards teen sex as one of numerous risk-taking behaviors constituting a "problem behavior syndrome" associated with a constellation of problem behaviors such as smoking, drinking, drug use, and delinquent behaviors constituting low-level status offenses [22]. Studies have found fairly consistent associations between externalizing problems such as conduct disorders (delinquency, aggressiveness, impulsiveness) and substance abuse (cigarette smoking, marijuana use, and use of other illicit drugs) and increased rates of early and high risk sexual behavior [29,32,33]. While associations between behavioral problems (aggression, delinquency) in childhood and increased risk of compromising sexual behaviors (including high rates of risky sex, frequent sexual activity, early sexual debut, low rates of condom use, high numbers of sexual partners, and high rates of prostitution and drug/alcohol use before and during sex [34]) has been well established in the literature, little is known about the factors and pathways that lead to such increased risk among adolescents who show signs of early conduct difficulties [35].

In contrast to robust associations between externalizing behaviors and adolescent sexual behavior, links between sexual behavior and internalizing factors have yielded mixed results [17,29]. On the one hand, a review of literature by [34] found that internalizing problems (low self-esteem, depression, and anxiety) are related to low perceived self-efficacy, which in turn is associated with decreased assertiveness, minimal ability to negotiate safe sex with a partner, sexually permissive attitudes, having sexually active friends, high risk of pregnancy, low contraception use, and non-virgin status. Other studies have specifically correlated depressive symptoms to high-risk sexual practices (such as early onset and contraception non-use) and negative health outcomes (such as unintended teenage pregnancy and contracting a sexually transmitted disease) [4,36]. On the other hand, other studies have found no significant effect between internalizing factors and risky sexual behavior in adolescence [29,34] or identified very limited effects of psychosocial predictors such as self-efficacy on sexual behavior [37]. Further definition of the relationship between mental health problems and adolescent sexual activity is vital because of the high rate of mental health problems which often take root in adolescence [38,39] and the opportunities for potentially effective interventions.

The hypothesis tested in this study is that sexual activity and high-risk sexual behavior in early adolescence (ages eleven to fifteen) is an expression of underlying psychosocial strains. We hypothesize that externalizing and internalizing psychopathology progressing from early middle school will be associated with higher rates of early and high-risk sexual activity. To test these hypotheses, we have divided the study into two parts. Our first study question examines the unique effects of internalizing psychopathology and externalizing psychopathology on initiation of sexual activity in middle school; our second study question examines the effects of these risk factors on high-risk sexual behavior. Distinct from other longitudinal studies on this topic, we also will examine how the rate of change in the risk factors over the course of middle school is associated with early and high sexual risk behaviors during middle school.

## Methods

This study is part of an ongoing project that aims to assess risk and protective factors for adolescent adjustment. In 2001 (Year 1), a survey was administered in a small northeastern city in the United States to all students in sixth grade (when students are usually eleven to twelve years old) in the public school system (n = 1,368) and was re-administered in 2002 (Year 2) and 2003 (Year 3), when most of the sample (96%) was in eighth grade (when students are usually thirteen to fourteen years old). (The remaining 4% had been retained a year and were in seventh grade (when students are usually twelve to thirteen years old).) To assess longitudinal changes in the variables of interest, only those students who completed the survey in both 2001 and 2003 were included in this study (n = 1,191). This attrition rate of 13% over the course of two years is characteristic of longitudinal studies with high-risk young, urban, ethnic-minority adolescents [40,41] such as in the present study.

Ethnicity was controlled for in all analyses and thus the sample was restricted to African-American, Hispanic, and Caucasian students. This resulted in the exclusion of sixteen (0.6%) subjects from other ethnic groups. The final working sample included 1,175 students. Analyses were conducted to determine whether the final sample of 1,175 students differed from the initial sample of 1,368 across 2001 study variables. Results indicated that the 193 students who dropped out did not differ from the students who remained in the study by race, gender, or any other variable of interest, except for their age (*t*(2, 1311) = 4.45, *p *= .000) and self-reported levels of violent delinquency (*t*(2, 1311) = 2.92, *p *= .004). Demographic characteristics of the final sample (N = 1,175) are presented in Table 1.

**Table 1:** Demographic characteristics of the study sample in sixth grade

*Variable*	*Total Sample *^a ^*N = 1,175*
*Age (Mean (SD))*	11.8 (0.72)
*Ethnicity*	
African-American	64.2%
Hispanic	26.4%
White	9.4%
Family structure	
Single parent	39.9%
Other	60.1%
*Mother's education *(High school or higher)	88.9%
*Father's education *(High school or higher)	89.9%
Lunch status	
Free	64.9%
Reduced Fee	9.0%
No	26.1%

^a ^Expressed as percent within group, unless noted otherwise

Eleven percent (n = 130) of all sixth grade students acknowledged having ever been sexually active in Year 1. These students were excluded from subsequent analyses. The prevalence of sexual initiation between Years 1 and 3, and of risky sexual behaviors in Year 3, is presented in Table 2.

**Table 2:** Sexual activity and risky sexual behaviors in the study sample

*Sexual activity*	*N = 1,175*
Active by sixth grade	11.1%
Active by eighth grade	21.0%
Not sexually active	67.9%
	
*Individual risky sexual behavior items (among those sexually active by eighth grade)*	*N = 247*
Not used condom last time had sex	7.2%
Had been drinking or using drugs	3.2%
No method used to prevent pregnancy	4.9%
How many times pregnant	
Once or not sure	3.7%
Two or more times	1.0%
Number of people had sex with	
1 person	41.9%
2–3 people	33.8%
4–5 people	9.8%
6 or more people	14.5%

### Procedure

The study was approved by the Yale School of Medicine Institutional Review Board and by the local Board of Education. Parents were informed of the survey at the time of school registration and offered the opportunity to decline participation. Prior to survey administration, students were read a detailed assent form outlining their participation with assurances of confidentiality and then asked for their signature to indicate assent (parent and child refusals were less than 1%). Surveys were group-administered to students in their classes by trained personnel affiliated with the school district and/or university. One administrator read surveys aloud to students while the students followed along. A second administrator was available for answering students' questions. Teachers remained in the classroom, but did not assist with the administration in order to protect the privacy of responses. The entire administration procedure typically lasted approximately one hour. Surveys were administered in English or Spanish, as appropriate, and a makeup administration day was scheduled for each school within one month of the initial administration for students who were absent. Lists provided by school principals were used to determine the language in which the survey was administered. Participants' scores did not systematically vary as a function of whether they spoke Spanish or English at home. Additional information about the procedure and measures has been described by [42] and by [43].

### Measures

The SAHA [44] represents a large-scale project on risk and protective factors for problem behaviors among inner-city youth. Detailed descriptions of the methodological aspects of the study are available in previous reports [20,42,45].

*(a) Sexual Activity *To assess sexual involvement, a dichotomous answer to the following question was used: *"Have you ever had sexual intercourse ('gone all the way')?"*

*(b) Risky Sexual Activity *was assessed using five individual indicators reflecting risky sexual behavior: "The last time you had sexual intercourse, did you or your partner use a condom?"; "The last time you had sexual intercourse, had you been drinking alcohol or using drugs?"; "The last time you had sexual intercourse, what method was used to prevent pregnancy?"; "How many times have you been/gotten someone pregnant?"; "With how many people have you had sexual intercourse?" The prevalence of risky sexual behaviors is presented in Table 2.

*(c) Socio-economic status (SES) *As a proxy for low SES, a composite index (0 to 6) was computed and consisted of single-parent family (0/1), parental level of education (lower than high school, calculated for each parent separately, 0 to 2), number of times the family moved during the two year period (3 or more times, 0/1), and child's free lunch status in school (no [0], reduced fee [1], free [2]).

(d) *Depressive symptoms *were assessed using an adaptation of the Center for Epidemiological Studies-Depression Scale (CES-D; [46]), which has demonstrated excellent psychometric properties with adolescents [47]. Students reported on the presence of symptoms during the past month using a three-point scale (Not True, Somewhat True, and Certainly True). The scale had good internal consistency (Cronbach alpha of 0.80 for both years).

(e) *Anxiety symptoms *were assessed by a 12-item scale [48] which included questions about worrisome, preoccupying thoughts or unpleasant feelings about self or external stimuli. The scale has good internal consistency (Cronbach alpha of 0.87).

*(f) Child Self-Report Post-Traumatic Stress Reaction Index (CPTS-RI) *is highly correlated with the DSM-based diagnosis of post-traumatic stress syndrome and designed to assess post-traumatic stress symptoms in school-aged children and adolescents after exposure to a broad range of traumatic events [49,50]. The Cronbach alpha for this scale was 0.86.

*(g) Problems Related to Substance Use *This scale consisted of five items developed by the SAHA Research Team [48] and asked whether the respondent had ever had problems related to the use of drugs (such as getting into an argument, feeling sick, getting arrested, or having financial problems). The scale had a Cronbach alpha of 0.73.

*(h) Antisocial Behavior Scales *[42] included two subscales assessing behavior problems of different severity. The *Non-violent Delinquency scale *consisted of five items describing non-violent antisocial behavior, such as stealing a car or pick-pocketing. The *Violent Delinquency scale *consisted of five items, pertaining to relatively serious aggressive and antisocial behaviors, including starting a fistfight, participating in a gang fight, hurting someone badly in a fight, and carrying a blade or knife to school. Coefficient alpha for these scales was 0.80 and 0.72, respectively.

### Statistical Analysis

#### Data Analysis Methods

Data were analyzed using the Statistical Package for the Social Sciences (SPSS, version 15.0) and HLM 6.0.

#### Psychopathology and Sexual Initiation

To examine the effects of internalizing and externalizing problems in sixth grade and their rate of linear change over the course of middle school on the likelihood of sexual initiation by Year 3 (when most participants were in eighth grade), a hierarchical binary logistic regression analysis was conducted. The regression analyses aimed to examine (1) demographic effects of gender, minority status and SES risk; (2) the direct effects of early levels of internalizing and externalizing problems (sixth grade), and (3) their estimated rates of linear change (slope) across the three waves of measurement. Multilevel modeling using HLM 6.0 was used to estimate individual participants' individual linear slopes based on three waves of data (Year 1, Year 2, and Year 3), and these estimates were outputted to SPSS for the regression analyses. Observed sixth grade scores were also included in the model.

#### Psychopathology and Risky Sexual Behavior

The second part of the analyses examined the association of externalizing and internalizing problems with risky sexual behavior in the subset of participants who did report sexual activity by the third year of the study (n = 235). For this analysis, hierarchical multinomial logistic regression was conducted with the risky sexual behaviors in Year 3 as the dependent variable. The independent variables for the second analysis were the same as in the prior set of analyses.

## Results

### Descriptive Results

Means and standard deviations for the measured variables are reported in Table 3. Additionally, the problems with substance use, nonviolent delinquency, violent delinquency, and risky sexual behavior variables were all substantially skewed. Although the sample size was large, violations of normality can be problematic in estimating rates of change using maximum likelihood estimation [51]. To reduce skewness and kurtosis, the natural logs of each of the externalizing variables were analyzed.

**Table 3:** Means and standard deviations of measured variables

	*N*	*Mean*	*SD*
Depression Yr 1	987	4.78	4.27
Depression Yr 2	896	4.10	4.25
Depression Yr 3	896	4.24	4.75
Anxiety Yr 1	988	10.78	5.63
Anxiety Yr 2	906	10.06	5.55
Anxiety Yr 3	890	9.59	5.46
Posttraumatic stress Yr 1	988	23.71	13.41
Posttraumatic stress Yr 2	909	21.20	13.05
Posttraumatic stress Yr 3	912	20.57	13.98
Problems with SU Yr1	1008	0.05	0.44
Problems with SU Yr 2	890	0.20	1.00
Problems with SU Yr 3	903	0.35	1.20
Nonviolent delinquency Yr1	1004	0.17	1.14
Nonviolent delinquency Yr2	894	0.47	1.94
Nonviolent delinquency Yr 3	896	0.81	2.81
Violent delinquency Yr 1	1001	0.86	1.69
Violent delinquency Yr 2	896	1.41	2.61
Violent delinquency Yr 3	896	1.90	3.49

Rates of linear change from Year 1 to Year 3 were estimated for each internalizing and externalizing factor in HLM 6.0 using full information maximum likelihood estimation. Individual HLM slope estimates were then exported to SPSS. The benefits of estimating rates of change in HLM rather than through OLS regression include more precise estimates and greater efficiency in dealing with data that may be missing in Year 2 [51,52]. Average slopes, as well as their correlations with measured sixth grade levels of internalizing and externalizing factors, are presented in Table 4. Each internalizing problem decreased significantly over the three years and each externalizing problem increased significantly over time. Furthermore, and not shown in the table, there was significant between-person variability in the slopes of each variable (*p *< .01), indicating individual differences in rate of change.

**Table 4:** Descriptive for estimates of slopes (*N *= 932)

*Variable*	*Slope*	*SE*	*r slope with sixth grade level*
Depression	-0.31**	0.08	-0.18**
Posttraumatic stress	-1.78**	0.23	-0.45**
Anxiety	-0.61***	0.10	-0.61**
Problems related to SU (log)	0.03**	0.003	0.10**
Nonviolent delinquency (log)	0.04**	0.005	0.18**
Violent delinquency (log)	0.06**	0.01	0.31**

* Significant at the 0.05 level (2-tailed)

** Significant at the 0.01 level (2-tailed)

### Psychopathology and Sexual Initiation

The first study question examined whether internalizing and externalizing symptoms in grade six, and rates of change in these symptoms over the course of middle school, would predict initiation of sexual activity by two years later, when most students were in eighth grade. Students who reported being sexually active by sixth grade were excluded from this analysis. Nine hundred thirty-two participants with full data on all measures were included in the analysis.

Students fell into one of two categories: those who reported being sexually active in Year 3 (n = 235 (23.5%)) and those who reported not being sexually active in Year 3 (n = 692 (74.6%)). The hierarchical logistic regression was conducted with sexual initiation (those students who were not sexually active in sixth grade, but reported becoming sexually active by the third year of the study, 1/0) as the dependent variable. Gender (male (1)/female (0)), race (with separate dummy variables created for African-American (1/0) and Hispanic (1/0) race), low SES, and sensation seeking were included as controls. Correlations among sixth grade predictor variables are reported in Table 5.

**Table 5:** Correlations among sixth grade variables (year 1;*N *= 932)

	*Male*	*African American*	*Hispanic*	*SES*	*Depression*	*Anxiety*	*Posttraumatic stress*	*Problems with SU (log)*	*Nonviolent delinquency (log)*
SES risk	-.02	-.05	.21**						
Depression	-.16**	-.09**	.10**	.07*					
Anxiety	-.12**	-.10**	.12**	.07*	.42**				
Posttraumatic stress	-.11**	-.01	.07*	.11**	.64**	.41**			
Problems with SU (log)	.08*	-.03	-.00	.00	.06	-.06	.13**		
Nonviolent delinquency (log)	.07*	.00	-.00	.03	.06	-.02	.09**	.31**	
Violent delinquency (log)	.20**	.11**	-.08**	.01	.12**	-.06	.15**	.24**	.30**

* Correlation is significant at the 0.05 level (2-tailed)

** Correlation is significant at the 0.01 level (2-tailed)

A hierarchical logistic regression was conducted to analyze the data. To facilitate comparison of odds ratios across independent variables, all continuous variables were converted to z-scores before being entered into the logistic regression so that the odds ratio of each could be interpreted using the same metric, which is the change in odds of initiating sexual behavior per increase of one standard deviation. Sixth grade levels of control variables and internalizing and externalizing factors were entered in the first hierarchical step of the logistic regression. Estimated slopes representing rates of linear change in internalizing and externalizing factors were imported from HLM entered in the second step of the logistic regression. The results from the final model are presented in Table 6, whereas the results from the initial step are described only in the text. In the first hierarchical step of sixth grade variables, gender, SES risk and sixth grade levels of violent delinquency were the only variables uniquely associated with increased risk of sexual activity by two years later. Males were almost twice as likely to initiate sexual activity over the course of the study, *odds ratio *= 1.99, p < 0.001 (95% CI: 1.44 – 2.73), and students with more SES risks were also more likely to initiate sexual activity, *odds ratio *= 1.22, *p *= 0.01 (95% CI: 1.04 – 1.42). Additionally, those who reported higher levels of violent delinquency in sixth grade were more likely to initiate sexual activity over the course of middle school, *odds ratio *= 1.26, *p *= 0.01 (95% CI: 1.06 – 1.51).

**Table 6:** Results from the final step of the hierarchical logistic regression predicting year 3 sexual initiation (*N *= 932)

*Variable*	*OR*	*CI*
Sixth Grade		
Male Gender	1.87**	(1.32–2.66)
SES risk	1.16	(0.98–1.36)
African-American	1.14	(0.64–2.05)
Hispanic	1.10	(0.59–2.06)
Depression	1.20	(0.95–1.53)
Anxiety	0.91	(0.72–1.16)
Posttraumatic stress	0.90	(0.68–1.17)
Problems with SU (log)	1.10	(0.89–1.37)
Nonviolent delinquency (log)	0.94	(0.75–1.20)
Violent delinquency (log)	1.05	(0.86–1.28)
Change		
Depression slope	1.09	(0.87–1.36)
Anxiety slope	0.79*	(0.64–0.99)
Post-traumatic stress slope	1.03	(0.80–1.31)
Problems with SU slope	1.27*	(1.04–1.56)
Nonviolent delinquency slope	1.21	(0.95–1.54)
Violent delinquency slope	1.55**	(1.26–1.91)

Continuous variables are standardized; odds ratios reflect one standard deviation increase.

CI = 95% confidence interval.

* Coefficient is significant at the 0.05 level (2-tailed)

** Coefficient is significant at the 0.01 level (2-tailed)

The addition of rates of change in Step 2 increased model fit, Δ*χ*^2^_(6*df*) _= 91.03, *p *< 0.01. The results from this step are presented in Table 6. There were unique effects of change in anxiety, substance use, and violent delinquency. Participants who experienced greater increases in anxiety over middle school were less likely to initiate sexual activity, *odds ratio *= 0.79, *p *= 0.04, (95% CI: 0.64 – 0.99). Participants who experienced greater increases in problems with substance use and violent delinquency were more likely to initiate sexual activity, *odds ratio *= 1.27, *p *= 0.02 (95% CI: 1.04 – 1.56), for substance use, *odds ratio *= 1.54, *p *< 0.001 (95% CI: 1.25 – 1.91), for violent delinquency. It should also be noted that in Table 6, sixth grade levels of violent delinquency no longer had a significant effect on increased likelihood of sexual initiation. This means that the effect of sixth grade levels was completely explained by the tendency for participants who reported more violent delinquency in sixth grade to grow more delinquent over time, *r *= 0.31, *p *< 0.001. Likewise, SES risk was no longer a significant predictor of sexual initiation in the final step of the analysis.

### Psychopathology and Risky Sexual Behavior

The second study question investigated whether internalizing and externalizing psychopathology were associated with risky sexual behavior among the participants who became sexually active during middle school. This part of the analysis was conducted with students for whom longitudinal data were obtained and who reported initiating sexual activity between Year 1 and Year 3. Two hundred thirty-five participants met the criteria for inclusion and had complete data on all measures. Of this sub-sample of sexual initiators, 34.8% reported engaging in no risky sexual behaviors, 38.9% reported engaging in one type of risky sexual behavior, 14.6% reported engaging in two types, 6.9% reported engaging in three types, and 4.8% reported engaging in four or more types of risky sexual behaviors. Given this distribution of risky sexual behaviors, hierarchical multinomial logistic regression analysis was used to examine the direct effects of the sixth grade variables (Step 1) and rates of change over the course of middle school (Step 2) on amount of risky sexual behaviors reported at Year 3. As in the prior set of analyses, continuous variables were standardized to facilitate interpretation of odds ratios. The risky sexual behavior variable was broken into three groups – no risks, one risk, and multiple risks – with no risks used as the reference group.

The results from the final model are presented in Table 7, whereas the results from the initial step are described only in the text. Of the sixth grade variables entered in Step 1, gender, African-American ethnicity, and problems with substance use each uniquely increased the fit of the model, *χ*^2^_(2*df*) _= 9.61, *p *< 0.01 for gender, *χ*^2^_(2*df*) _= 10.25, *p *< 0.01 for African-American, and *χ*^2^_(2*df*) _= 6.94, *p *= 0.03 for problems with substance use. Males were almost three times as likely to engage in one risky sexual behavior, *odds ratio *= 2.83, *p *= 0.004 (95% CI: 1.40 – 5.73), but were no more likely to engage in multiple risky sexual behaviors, *odds ratio *= 1.18, *p *= 0.66 (95% CI: 0.56 – 2.45). Participants with more problems with substance use in sixth grade were significantly more likely to engage in one risky sexual behavior, *odds ratio *= 1.71, *p *= 0.04 (95% CI: 1.02 – 2.89), but were no more likely to engage in multiple risky sexual behaviors, *odds ratio *= 1.04, *p *= 0.89 (95% CI: 0.57 – 1.91). Although the African-American variable contributed uniquely to model fit, comparisons indicated that African-Americans were not significantly more or less likely to engage in one or multiple risks.

**Table 7:** Results from the final step of the multinomial logistic regression predicting risky sexual behaviors at year 3 (*N *= 235)

	*Overall*	*One risk*		*Multiple risks*	
*Variable*	*χ*^2^_(2*df*)_	*OR*	*CI*	*OR*	*CI*

Sixth Grade					
Male Gender	9.55**	3.15**	(1.50–6.64)	1.69	(0.73–3.90)
SES-risk	0.54	1.13	(0.82–1.56)	1.05	(0.74–1.49)
African-American	3.72	2.86	(0.73–10.94)	0.89	(0.25–3.25)
Hispanic	0.07	1.10	(0.28–4.34)	0.92	(0.25–3.37)
Depression	2.60	0.74	(0.45–1.22)	0.67	(0.39–1.14)
Anxiety	0.59	0.95	(0.56–1.60)	0.80	(0.45–1.43)
Posttraumatic stress	6.13*	1.31	(0.72–2.36)	2.15*	(1.15–4.00)
Problems with SU (log)	5.98*	1.73*	(1.03–2.93)	1.16	(0.62–2.17)
Nonviolent delinquency (log)	5.21	0.63	(0.40–1.01)	0.63	(0.37–1.09)
Violent delinquency (log)	0.76	1.18	(0.81–1.72)	1.13	(0.74–1.73)
Change					
Depression slope	1.49	1.09	(0.69–1.72)	1.33	(0.83–2.13)
Anxiety slope	2.22	0.77	(0.48–1.22)	0.70	(0.42–1.16)
Post-traumatic stress slope	1.74	1.14	(0.66–1.96)	1.42	(0.83–2.45)
Problems with SU slope	2.23	1.12	(0.76–1.67)	1.34	(0.90–1.98)
Nonviolent delinquency slope	0.39	0.89	(0.57–1.39)	1.01	(0.64–1.59)
Violent delinquency slope	2.33	1.22	(0.82–1.82)	1.40	(0.90–2.17)

Reference group is 'no risks.' Continuous variables are standardized; odds ratios reflect one standard deviation increase. CI = 95% confidence interval.

* Coefficient is significant at the 0.05 level (2-tailed)

** Coefficient is significant at the 0.01 level (2-tailed)

The inclusion of the rates of change estimates in the second step of the model increased model fit, Δ*χ*^2^_(12*df*) _= 28.88, *p *< 0.05. The results of the final model are presented in Table 7. As indicated in the table, even though the inclusion of the block of rates of change variables increased overall model fit, none of the unique effects of rates of change was statistically significant. The effects of gender and sixth grade problems with substance use were maintained, and the pattern of effects was similar to that in Step 1: males were more likely to engage in one risky sexual behavior, but were not more likely to engage in multiple risky sexual behaviors. Similarly, participants with more problems with substance use were significantly more likely to engage in one risky sexual behavior, but were not significantly more likely to engage in multiple risky sexual behaviors.

## Discussion

This study examined the unique effects of several forms of internalizing and externalizing psychopathology on the likelihood of initiating sexual activity and engaging in unsafe sex in middle school. Utilizing a three-year longitudinal design with three waves of measurement, we included sixth grade levels of psychopathology and the rate of change in psychopathology over the course of middle school as predictors of sexual initiation and high-risk sexual behavior.

In our study, males were twice as likely as females to initiate sexual intercourse early and three times as likely to engage in high-risk sexual behaviors as compared to girls of the same age. This discrepancy matches that seen in national statistics which show greater than two-fold higher rates of sexual activity among young teenage boys as compared to young teenage girls [53]. It may be that males are more likely to report such behaviors than girls, rather than more likely to in fact engage in such behaviors.

We also found that respondents of lower socio-economic status (which included family structure, parental education, and a proxy measure for economic status), had an increased risk of initiating intercourse earlier than their peers. This finding is consistent with previous research which has found that adolescents from families with single parents, of lower income and/or lower parental education have an earlier age of sexual initiation than their peers [54]. Some have hypothesized that poverty, with which single-parent and families with low parental education are associated, increases the likelihood of adolescent risk behaviors because of limited and low-quality social and educational resources in low-income neighborhoods and economic stress leading to lower parental supervision [55]. Although lower socio-economic status was initially related to increased risk of sexual initiation, this association dropped from significance when psychopathology change-over-time variables were entered into the analysis, most likely because socio-economic status is also associated with greater increases in externalizing factors and smaller decreases in internalizing problems.

Recent studies of American teenagers have found that younger age of sexual initiation is correlated with higher sexual risk behaviors such as increased numbers of sexual partners and lower levels of contraceptive use [5,7], which in turn is associated with increased risk for unintended pregnancies and contracting sexually transmitted diseases [8]. Given the trend towards younger age of sexual initiation, gearing programs for adolescent sexual health towards young adolescents, and tailoring these efforts to gender-specific needs, is warranted. Further, providing follow-up guidance and care for those already active at younger ages may reduce the negative impacts seen from earlier sexual initiation.

Of the psychosocial factors examined, we found that externalizing factors are more predictive of sexual risk in early adolescence than are internalizing factors. This conclusion supports similar findings in other literature documenting associations between childhood externalizing disorders and deviant problem behaviors ([56,57] in [21]) such as a paper by [21] which found childhood externalizing psychopathology to be a more robust prospective predictor than internalizing psychopathology of early onset substance abuse behaviors. As a whole, the concurrence of sexual risk behaviors with substance use and mental health problems suggests that interventions around sexual health should be multi-dimensional (e.g. address substance use and mental health well-being) rather than only focusing on sexual behaviors or attitudes [58].

Of the externalizing factors studied, increasing incidence of violent behavior (such as starting a fistfight, participating in a gang fight, hurting someone badly in a fight, and carrying a blade or knife to school) and increasing abuse of substances during middle school heightened the risk of sexual onset by eighth grade (OR 872.79 and OR 59.69 respectively). In contrast, only sixth grade problems with substance use forecasted increased likelihood of engaging in at least one risky sexual behavior by the end of middle school (OR 186.40), whereas neither nonviolent nor violent delinquency was uniquely associated with high-risk sexual behavior. The associations of increasing violence and substance abuse with the onset of early sexual activity and association of early substance abuse with high-risk sexual behavior suggest that programs to stem violence and substance abuse early on may have an added effect of delaying sexual onset and reducing high-risk sexual behavior in early teen years.

The associations between externalizing behaviors and risky sexual behaviors among teenagers corroborates with the Jessor Problem-Behavior Theory that engaging in risky sexual behavior (early initiation, involvement in one or more risky behaviors) is part of a syndrome of problem behaviors. According to the Jessor Theory, the likelihood of engaging in problem behavior depends on personality characteristics (such as low expectations for academic achievement and high tolerance of deviance), social environmental factors (such as parenting style and peer influences), and other behaviors (such as low school achievement) that reflect greater or lesser orientation toward, attachment to, and involvement with conventional values, goals and institutions [30]. Various hypotheses exist to explain these correlations, though full discussion of these is beyond the scope of this paper. For example, in terms of personality characteristics, it has been postulated that this syndrome of deviant behaviors stem from a common source such as low self-control [22]. In terms of social environmental factors, [59] stress the influence of peer factors, hypothesizing that early participation in minor deviant behaviors such as alcohol and tobacco use result in separation from conventional peer influences and engagement in associations with friends who are already participating in other types of adult behaviors. In terms of substance abuse and sexual risk behavior, researchers have theorized that teens who use drugs or alcohol are also more likely to be sexually active at earlier ages possibly due to the disinhibiting effects of these substances on adolescents' decisions to delay intercourse [4].

Associations between these internalizing factors and sexual behavior are less robust than externalizing behaviors, consistent with findings in the literature. Of the internalizing psychosocial factors examined, we found that students who reported greater increases in anxiety symptoms were less likely to initiate sexual activity over the course of middle school. However, in contrast to the findings in this analysis, a study by [60] found that adolescents who report high levels of anxiety or stress are more likely to have multiple sexual partners and less likely to use condoms than those with lower levels of anxiety and stress [61]. Thus anxiety may limit risk-taking behaviors, or alternatively, the intimacy of intercourse may be seen as a means to release anxiety. Further definition of the anxiety may help to clarify this relationship and tease apart higher versus lower-risk teens.

Additionally in this study, neither symptoms of depression nor posttraumatic stress were uniquely related to sexual initiation or high risk sexual behavior. In contrast, studies which have found that depression leads to higher risk of early sexual activity such as by [17,26,62] and [63] theorize that distant or low-quality relationships with parents result in depressed emotional states that increase vulnerability to peer influence and peer support, which in turn may make adolescents more susceptible to engaging in early intercourse. It may be that internalizing factors moderate sexual behaviors differently depending on the age and developmental stage of the adolescent, as has been demonstrated in studies of psychopathology and substance abuse in teenagers. For example, [21] showed that studies correlating psychopathology to substance use found that later onset alcoholism was related to internalizing psychopathology, whereas earlier onset alcoholism was more strongly related to general disinhibition and novelty seeking. These researchers concluded that an internalizing pathway for substance problems may not be operating until late adolescence [21]. A recent longitudinal study by [39] found that middle school and high school boys and girls with high depressive symptoms at baseline were significantly more likely than those with low depressive symptoms to report at least one sexual risk behavior during the follow-up period. However, the study did not report on how age may moderate these effects. Future longitudinal studies which stratify by age group may help to clarify how age may moderate the relationship between internalizing factors and adolescent sexual behavior.

### Study Limitations

This study benefited from high participation rates, inclusion of both boys and girls, and longitudinal methodology with matching of questionnaires from initial and later data points. However, several limitations related to challenges in measuring sexual behaviors and psychosocial factors for young teens should be noted. Given that results were drawn from self-report surveys filled out by adolescent study participants, conclusions about the results may be constrained by cognitive limitations, recall bias, reporter bias, and social desirability bias [64]. For example, young participants may have difficulty understanding the questions asked or responding to questions which are beyond their level of experience [12]. Participants may also alter their responses based on perceived peer norms and concerns about confidentiality. While studies have shown that the majority of respondents maintain consistency in reporting sexual behaviors over time [65,66], a study by [67] found the greatest inconsistencies in reporting among young teen African-American boys. The conclusions that can be drawn from this study are also limited in that participants were asked only about "sexual intercourse (going all the way)" and not about engaging in other specific sexual behaviors such as oral or anal sex which may not be considered "intercourse" per se, but are considered high-risk behavior for STD transmission [68]. The findings from this study may be generalizable to only those adolescents with similar demographic characteristics, namely being minority and inner-city youth in the United States in this era. Also, whereas externalizing behaviors generally ask about definitive events (ever smoked before), the equivocal symptoms of internalizing factors (symptoms over the past month) may limit the ability of a short survey to correlate emotional states with sexual behaviors. Qualitative studies may provide a more comprehensive understanding of these relationships. Finally, as suggested by [19], future studies may benefit by including psychosocial factors in a risk and protective model predicting risky sexual behavior among young adolescents.

## Conclusion

Understanding psychosocial factors associated with early and high-risk sexual activity among young adolescents has implications for prevention programs and public policy which aim to mitigate negative consequences of adolescent sexual activity.

### Importance of early interventions

National studies have focused on sexual activity of high school students, but studies at the middle school level have only been done by a handful of cities and districts around the country. More comprehensive studies of this younger age group may help identify additional risk factors and outcomes associated with sexual behavior specific to the developmental stage of middle school students. [69] advocate that clinicians begin screening and counseling for risk behaviors in early adolescence (e.g. late elementary and middle school). Furthermore, the finding that those who show increasing violent delinquency and greater substance abuse over the course of middle school are more likely to engage in sexual activity in these early years suggests that intervening early on may help to stem these risky behaviors.

### Importance of addressing psychosocial needs associated with early and high risk sexual behavior

The results suggest that externalizing psychopathology is a more consistent predictor of early and risky sexual behavior than is internalizing psychopathology. In a review of the past decade of adolescent STI/HIV interventions, [64] found that tailoring interventions to target populations are markedly more effective in reducing behaviors which increase risk of contracting a sexually transmitted disease. Children with more externalizing psychopathology may be a higher-risk group for negative consequences of risky sexual behavior, and thus a key group on which to focus sexual risk reduction programs. In this study, adolescents who show signs of violent delinquency and substance abuse are most likely to engage in sexual risk behavior by the end of middle school. Consequently, sexual health programs for young adolescents may benefit from addressing the externalizing behaviors themselves. For example, sexual education programs might incorporate violence reduction strategies. Conversely, violence reduction programs to identify and intervene with those adolescents engaging in violent behaviors may serve as an additional way to delay sexual onset and reduce the risk of risky sexual activities. Additionally, programs which effectively reduce alcohol and drug use may have additional value in delaying the initiation of sexual intercourse [12]. Ultimately, as suggested by [58], sexual education programs can benefit from a multi-dimensional approach that addresses emotional and behavioral well-being in addition to sexual behavior and attitudes.

## Authors' contributions

A. Caminis researched the background literature on related subject material and coordinated the manuscript draft. C. Henrich carried out biostatistical analyses. V. Ruchkin carried out biostatistical analyses and was instrumental in designing and administering the survey tool. M. Schwab-Stone participated in the design of the original study and was also instrumental in designing and administering the survey tool. A. Martin participated in the study design and helped to coordinate the manuscript draft. All authors read and approved the final manuscript.
